# Genome-wide identification of MADS-box family genes in moso bamboo (*Phyllostachys edulis*) and a functional analysis of *PeMADS5* in flowering

**DOI:** 10.1186/s12870-018-1394-2

**Published:** 2018-09-03

**Authors:** Yuting Zhang, Dingqin Tang, Xinchun Lin, Mingquan Ding, Zaikang Tong

**Affiliations:** 1 State Key Laboratory of Subtropical Silviculture, Zhejiang A & F University, Lin’an, Zhejiang China; 2 The Key Laboratory for Quality Improvement of Agricultural Products of Zhejiang Province, College of Agriculture and Food Science, Zhejiang A & F University, Lin’an, Zhejiang China

**Keywords:** MADS-box, Bamboo, Gene expression pattern, Flower time, Floral organ

## Abstract

**Background:**

MADS-box genes encode a large family of transcription factors that play significant roles in plant growth and development. Bamboo is an important non-timber forest product worldwide, but previous studies on the moso bamboo (*Phyllostachys edulis*) MADS-box gene family were not accurate nor sufficiently detailed.

**Results:**

Here, a complete genome-wide identification and characterization of the MADS-box genes in moso bamboo was conducted. There was an unusual lack of type-I MADS-box genes in the bamboo genome database (http://202.127.18.221/bamboo/index.php), and some of the *PeMADS* sequences are fragmented and/or inaccurate. We performed several bioinformatics techniques to obtain more precise sequences using transcriptome assembly. In total, 42 MADS-box genes, including six new type-I MADS-box genes, were identified in bamboo, and their structures, phylogenetic relationships, predicted conserved motifs and promoter *cis*-elements were systematically investigated. An expression analysis of the bamboo MADS-box genes in floral organs and leaves revealed that several key members are involved in bamboo inflorescence development, like their orthologous genes in *Oryza*. The ectopic overexpression of one MADS-box gene, *PeMADS5*, in *Arabidopsis* triggered an earlier flowering time and the development of an aberrant flower phenotype, suggesting that *PeMADS5* acts as a floral activator and is involved in bamboo flowering.

**Conclusion:**

We produced the most comprehensive information on MADS-box genes in moso bamboo. Additionally, a critical *PeMADS* gene (*PeMADS5*) responsible for the transition from vegetative to reproductive growth was identified and shown to be related to bamboo floral development.

**Electronic supplementary material:**

The online version of this article (10.1186/s12870-018-1394-2) contains supplementary material, which is available to authorized users.

## Background

MADS-box genes encode a large family of transcription factors that have essential roles in animals, plants, and fungi [1]. The first plant MADS-box genes were found to regulate floral meristem identity. Since then, several MADS-box genes have been reported to control the vegetative to reproductive phase transition in plants and developmental processes in plant organs, such as fruit, root, stem, and leaf [2–4]. This large gene family contains two groups, type I (SRF-like) and type II (MEF2-like), based on their conserved domains [5]. In plants, the type-I genes encode plant-specific transcription factors of the Mα, Mβ, and Mγ subfamilies, while the most well-known MADS genes are from the type-II group, such as the MIKC^C^-type and MIKC*-type [6, 7]. The term MIKC originated from the four major domains of proteins encoded by type-II genes: the MADS (M) domain followed by an Intervening (I) domain, a second most conserved Keratin (K) domain and a C-terminal domain [8].

Flowering is a complicated process that requires cooperation and interaction among numerous genes. Loss-of-function plant mutations have been used to identify many MADS-box genes that play crucial roles in flowering processes, including flowering time (SUPPRESSOR OF OVEREXPRESSION OF CONSTANS1 (SOC), FLOWERING LOCUS C (FLC), and SHORT VEGETATIVE PHASE (SVP)) [9–11], formation of floral meristems and organs (*APETALA1* (AP), *FRUITFULL* (FUL), *PISTILLATA* (PI), *APETALA3* (AP_3_), *SHATTERPROOF* (SHP) 1/2, *SEPALLATA* (SEP) 1/2/3 and *AGAMOUS* (AG)) [12–16], fruit ripening (SHP1 and SHP2) [17], seed pigmentation, and embryo development (TRANSPARENT TESTA16) [8, 18]. The ABCDE model explains the ontogeny of dicot flowers [16, 19]. In general, A-class genes participate in sepal development, whereas C-class genes are involved in carpel development. Petal development requires both A- and C-class genes, whereas stamen development needs both B- and C-class genes. D-lineage genes are necessary for ovule development [15, 20, 21]. E-class genes redundantly specify all floral organ identities and floral meristem determination [16]. Almost every gene from this model, except *Arabidopsis AP*_*2*_, belongs to the type-II MADS-box subfamily, indicating that MADS-box genes play pivotal roles during flowering. Compared with type-II MADS-box genes, type-I genes in plants have attracted less attention [22]. However, functional studies of type-I MADS-box genes have revealed their crucial roles during plant reproduction and development, especially in determining female gametophyte, embryo, and endosperm development in *Arabidopsis* [22].

Bamboo is an important non-timber forest product worldwide. The plant is beneficial to the human food supply and several industries, as well as to the conservation of the environment and animal habitats. Moso bamboo (*Phyllostachys edulis*), native to China, is a large woody bamboo species with a complex rhizome system, hollow and highly lignified culms, and bisexual spikelets [23]. For the many years of the long vegetative phase, moso bamboo culms (building materials) and young shoots (edible vegetable) can be continually harvested. However, moso bamboo only flowers once and dies after seed production (monocarpy) [24]. Woody bamboos typically exhibit synchronous flowering in a community in which all culms flower and die within the same year [25]. The switch from vegetative to reproductive growth is difficult to predict. These unique characteristics have led to centuries of study by scientists and enthusiasts. Several hypotheses have been proposed for the mechanism of bamboo flowering, and physiological and biochemical studies have been conducted [23]. External controls, such as photoperiod and drought cycles, may initiate flowering [26, 27], and endogenous factors may control flowering induction, including the circadian clock, which has been supported by observations of synchronous flowering in parental stocks and transplanted seedlings [28, 29]. However, the mechanism underlying the molecular regulation of flowering remains unclear.

In eudicots, the expression of some MADS-box genes can affect flowering time and the development of floral structures, which make MADS-box genes good candidates to understand the unique flowering patterns in bamboos. Lin identified two AP/SQUA-like MADS-box genes from *Phyllostachys praecox*, *PpMADS1* (FUL3 subfamily) and *PpMADS2* (FUL1 subfamily) [30]. Both genes play vital roles in the floral transition of bamboo. Next-generation sequencing allows the investigation of functional genes and flowering pathways on a genome scale. In the caespitose bamboo *Bambusa edulis*, two sequencing platforms have been used and 16 MADS-box genes (*BeMADS*) identified [31]. Most *BeMADS* genes are highly expressed in floral organs and share similar expression patterns with their homologs in *Oryza sativa*. A transcriptome analysis in *P. edulis* identified 38 putative MADS-box transcription factors in panicle tissues [32]. Although the first version of the bamboo genome sequence is available, the annotations of several genes are still tentative owing to the limitations of the previously used sequencing and assembling technologies. Here, we report a novel and systematic study of the MADS-box gene family involving not only database sequence identification but also sequence complementation and correction. We identified 42 MADS-box genes and then investigated the structures, phylogenetic relationships, conserved protein motifs, duplications, and expression patterns of these genes during floral development in bamboo. The overexpression of an *AGL24* homologous gene, *PeMADS5*, in *Arabidopsis* resulted in early flowering and floral abnormalities. Yeast two-hybrid analyses revealed that PeMADS5 can interact with PeAP1 (PeMADS2) and PeSOC1 (PeMADS34), which suggests that PeAGL24 and PeSOC1 act as dimers to promote flowering. This study will serve as a useful reference for further functional analyses of candidate genes involved in bamboo floral development.

## Methods

### Identification and classification of MADS-box genes

We downloaded *P. edulis* genome sequences from the Bamboo Genome Database (http://202.127.18.221/bamboo/index.php). MADS-box protein sequences from *Arabidopsis*, *Oryza,* and *Brachypodium* were obtained from published studies [8, 18, 33]. To acquire the maximum number of MADS-box domain containing sequences in moso bamboo, we built three different Hidden Markov Models (HMM) profiles to search the *P. edulis* protein dataset based on *Arabidopsis*, *Oryza,* and *Brachypodium*, respectively. Gene name query was also employed on NCBI and BambooGDS. All of these candidate MADS-box genes were checked manually to remove the incomplete and redundant sequences. The nomenclature of putative type II bamboo MADS-box genes was assigned based on their scaffolds rankings.

### Determination of presence and absence of type I MADS-box gene clades

In the first round of identification, type I MADS-box subfamily genes escaped detection in the HMM search. Therefore, the coding sequences (CDS) of Type I MADS-box from *Oryza* and *Brachypodium* were used as the probe sequences to blast against the bamboo genome as local databases. We obtained the best three BLAST results to check whether they represent MADS-box genes that were already in our dataset. If not, the CDS and protein sequences were predicted by Fgenesh ++ software [34] based on the corresponding genomic scaffold.

### Correcting the incomplete *PeMADSs* sequences

The *P. edulis* transcriptome reads were downloaded from the NCBI SRA database (Accession: SRR4450542, SRR4450543, SRR4450544, SRR4450545, SRR4450546, SRR4450547, SRR4450548, SRR4450549, SRR4450550, SRR4450551). One of the methods to correct *PeMADSs* sequences was using Trinity software. After trimming low-quality sequences, transcriptome data was de novo assembled with the Trinity software using default parameters [35]. Because we are only interested in the *PeMADSs,* all of the CDS sequences of the MADS-box family genes were used as the references. Then the software of the Tablet was employed to exhibit the alignment results [36]. To identify the incomplete 5′ and 3′ end sequence of the genes, another method to complete the sequences was using in-house scripts to conduct e-Genome-walking process. To validate these complementary sequences, full-length CDS sequences of 16 *PeMADSs* were amplified (Primers in Additional file 1: Table S1) and cloned into pMD18-T vectors. After sequencing, multiple sequence alignments among the cloned, assembled and original sequences were conducted to explore the accuracy of the methods we used here.

### Subcellular localization analysis

The coding sequences of *PeMADS23* and PH01002755G0230 without the stop codon were amplified and then subcloned into the p2GWY7 vector and fused in-frame with the Yellow Fluorescent Protein (YFP) sequence under the control of the *CaMV35S* promoter. The fusion constructs were introduced into *Arabidopsis* protoplasts prepared from 4-day suspension cells by using 40% polyethylene glycol (PEG) as described previously [37]. YFP fluorescence was observed with a laser scan confocal microscope. The transient expression assay was repeated three times.

### Phylogenetic analysis

*P. edulis* MADS-box proteins were aligned with *Oryza*, *Arabidopsis* by MAFFT [38]. The alignment was cropped to remove low conserved regions and finally contained the M- and part of the K domain. An unrooted neighbor-joining (NJ) tree was constructed using the MEGA7 package [33, 39]. The tree nodes were evaluated by bootstrap analysis for 1000 replicates. Branches with less than 50% bootstrap values were collapsed.

### Conserved motif analysis

The conserved motifs were investigated by MEME version 2.2 online tool (Multiple Expectation Maximization for Motif Elicitation) (http://meme-suite.org/) [40]. The parameters are set as follows: number of repetitions: any, the maximum number of motifs: 10, optimum motif width set to≥6 and ≤ 200. The motifs obtained were annotated using the Simple Modular Architecture Research Tool (SMART) and NCBI CD search program [41, 42].

### Cis-element enrichment analysis

PlantCARE database (http://bioinformatics.psb.ugent.be/webtools/plantcare/html/) was used to predict cis-regulatory elements in the *PeMADS* promoter (1.5 kb upstream from the translational start codon) and intron region [43]. In this study, we selected cis-element associated with core promoter elements, protein binding sites, hormones responses, tissue-specific elements, light responsive elements, abiotic and biotic stress responses, circadian responses and cell cycle regulation elements.

### Collection of plant material

The inflorescence samples of flowering moso bamboo and the leaf samples from non-flowering plants were collected in Guilin, Guangxi province from June to July 2016. The identification of four stages of bamboo inflorescence development is based on the numbers of florets and anatomical structures (stages F1–4): the first floral bud formation, initial stage of inflorescence development (3–5 florets), maturation of inflorescence (~ 10 florets) and anthesis. The leaf tissues were collected from the non-flowering moso bamboo, under the same growth environment with the flowering plants. The panicle and leaf samples were frozen in liquid nitrogen immediately and stored at − 70 °C. Total RNA was extracted using the RNAprep pure Plant Kit (TianGen, China). Contaminating DNA was removed using DNase (TaKaRa Bio Inc., Japan). After checking the quality of purified RNA on an agarose gel, the RNA samples were quantified by NanoDrop ND-1000 spectrophotometer (NanoDrop Technologies Inc., USA). RNA was reverse transcribed from 5 μg of total RNA in 100 μL of reaction volume using the PrimeScript™ RT reagent kit (TaKaRa Bio Inc., Japan) according to the manufacturer’s instructions. The resulting cDNA was used for further experiments.

### Expression analysis of MADS-box genes

The primers used for MADS-box expression analysis were designed by Primer Premier 5 (Additional file 1: Table S2). The expression levels of target genes were detected with SYBR® Premix Ex Taq II (TaKaRa Bio Inc., Japan). All qPCR assays were carried out in a CFX- 96-well Real-Time System (BioRad, USA). The reaction mixture consisted of 10 μL SYBR Green mix, 200 nM of each primer, and 2 μL of diluted cDNA in a final volume of 20 μL. The qPCR protocol consisted of an initial thermal cycling step of 95 °C for 3 mins, followed by 40 cycles of denaturation at 95 °C for 10 s and annealing with a temperature gradient from 50 to 60 °C for 30 s. All experimental samples were repeated in triplicate. To normalize the variance among different samples, NTB was used as a housekeeping gene for data normalization [44, 45]. The raw Cycle threshold (Ct) values were calculated automatically by the Bio-Rad CFX Manager (version 2.3) using the 2^-△△ct^ method [46].

### Construction of *PeMADS5* ectopic expression transgenic *Arabidopsis* lines

The full open reading frame of the *PeMADS5* was amplified from cDNA using the primer pairs *PeMADS5* (Additional file 1: Table S1) and cloned into pMD18-T Vector. The pCXSN (FJ905214) vector containing the CaMV*35S* promoter and the *Nos* terminator was digested with *Xcm*I (New England Biolabs) [47]. The PCR product was amplified from pMD18-T followed by an A-addition procedure. The digested pCXSN plasmid and the PCR-amplified product were ligated using T4 DNA ligases (NEB). All recombinant plasmids identified from each individual *E coli* colone were verified by sequencing. This ectopic expression construct was named *35S::PeMADS5*. Healthy wild-type *Arabidopsis* (Columbia-0) plants were grown on soil under long-day photoperiod conditions (16 h of light/8 h of darkness). The construct was transformed to *Agrobacterium tumefaciens* strain GV3101 and used to transform *Arabidopsis* by the floral dip method [48]. *Arabidopsis* seeds obtained after transformation were plated on one-half-strength Murashige and Skoog medium containing 30 mg hygromycin for selection. Rosette leaves of Col-0, 35S::*PeMADS5* #2 and #10 were obtained when plants bore a 1-cm-long inflorescence. The inflorescences from col-0 and transgenic plants were also collected on full flowering stage. Plant materials were sampled for qRT-PCR. Statistical analysis for the results from qPCR expression data and bolting days of *Arabidopsis* were carried out using a Student’s t-test. All data are presented as mean ± SD. *p* ≤ 0.05 and *P* ≤ 0.01 were considered statistically significant compared with the Columbia-0 (Col-0) wild-type.

### Yeast two-hybrid assay

The experimental procedures of yeast two-hybrid assay were performed using the Matchmaker Two-Hybrid System (Clontech). The full coding sequence of *PeMADS2, 5, 16*, *20* and *34* were cloned into both pGBKT7 and pGADT7 vectors. The resulting recombinant plasmids were introduced into yeast strains Y2HGold and Y187, respectively. Two-hybrid interactions were assayed on selective SD/−Trp/−Leu double-dropout (DDO). Potential interactions were tested on selectiveSD/−Trp/−Leu/-His/−Ade/X-α-gal (40 mg/ml) media supplemented with 5 mM 3-amino-triazole (3-AT).

## Results

### Identification and replenishment of *P. edulis* MADS-box genes

**Fig. 1:**
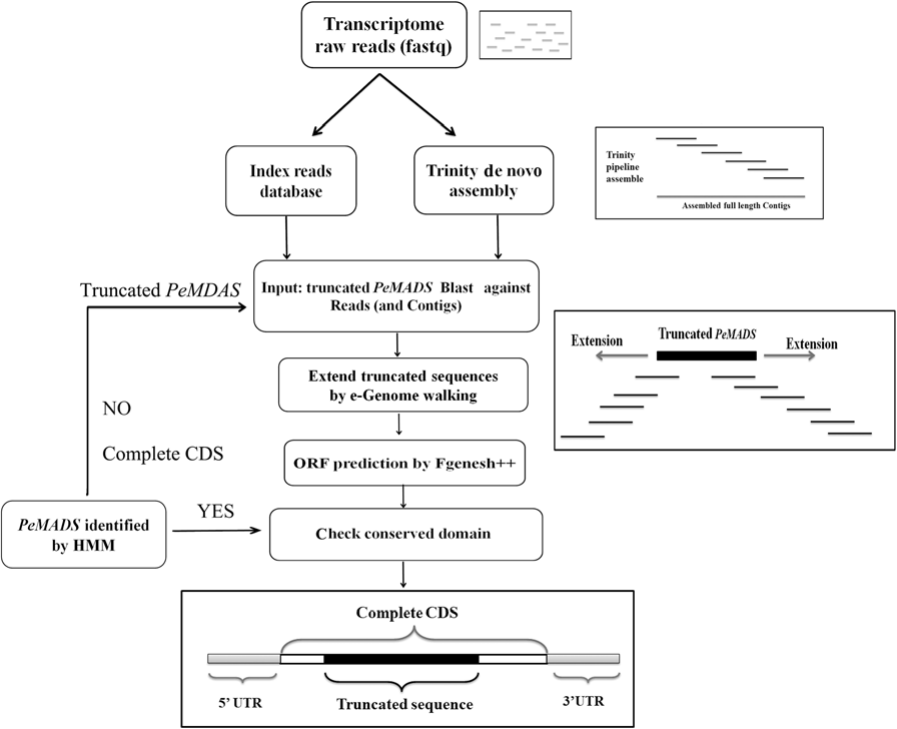
Schematic diagram of the bioinformatics strategy of the replenishment of *PeMADS* sequences

**Fig. 2:**
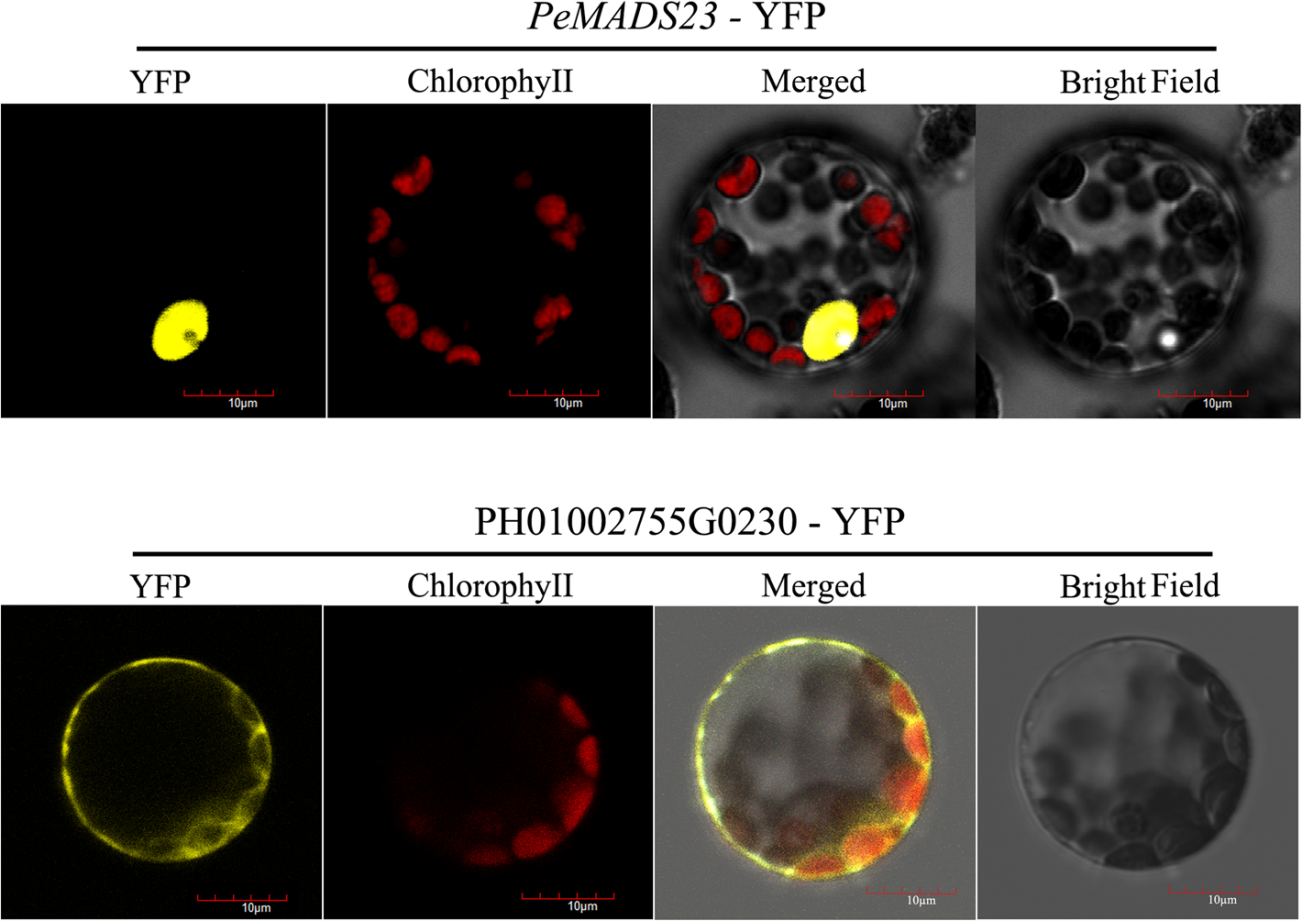
Subcellular localization analysis of PeMADS23 and PH01002755G0230. Constructs of PeMADS23-YFP and PH01002755G0230-YFP were transfected into *Arabidopsis* protoplasts. The fluorescence signal was detected with a laser scanning confocal microscope. YFP indicates fluorescence of YFP, and the red color shows the auto-fluorescence of chlorophyll. The length of the bar is 10 μm

The MADS-box protein sequences of three species, *Oryza*, *Arabidopsis*, and *Brachypodium*, were used to build a hidden Markov model, which was used to search the moso bamboo protein dataset. In total, 36 unique MADS-box proteins were identified (Table 1). *PeMADS*s (*PeMADS18, − 27, − 30, − 36*, − *37*, − *38*, − *42*, and − *44*) do not contain the most conserved MADS domain, and they were verified to be pseudogenes. Furthermore, five type-I genes, a PI-like clade gene (*PeAP3*) and an AGL6-like clade gene (*PeAGL6*) were obtained by running BLAST algorithm-based searches of the Bamboo genome databases with *Oryza* and *Arabidopsis* homologous genes. In total, 42 *MADS* genes have been identified in *P. edulis.* However, owing to the limited accuracy of *Phyllostachys* scaffolding, some *PeMADS*s may lack full-length gene sequence information. Thus, we developed a bioinformatics pipeline to recover the full-gene regions using the transcriptome sequencing data. First, the transcriptome reads were indexed as the references, and then, the incomplete *PeMADS* sequences were used as query in BLAST algorithm-based searches of the reads database. The optimally aligned reads were chosen to extend the open reading frame of each gene. The newly modified sequences were then included in a new search cycle until no new reads could be identified for any extension. All of the replenished coding DNA sequences (CDSs) were translated by Fgenesh++ software (Fig. 1) [34]. A total of 17 *PeMADS* genes were further corrected or completed (Table 2). Among them, 13 *PeMADS* genes lacked the M- or K-domain, while four other genes have incorrect sequences in their open reading frame regions. After correction, the *PeMADS* sequences’ quality and integrity were greatly improved. For instance, a K-domain, which is one of the most conserved domains in MADS-box genes, was added to *PeMADS23* (Additional file 2: Figure S1). These 17 full-length *PeMADS* genes were also experimentally cloned from *P. edulis* cDNA with gene-specific primers to further confirm the sequence accuracy (Additional file 1: Table S1). To determine the accuracy levels of the modified sequences, we chose one gene, *PeMADS23*, to undergo a functional analysis. Using a subcellular localization prediction tool, *PeMADS23* (corrected sequence) was found to have a nuclear subcellular location signal that is not found in the original sequence PH01002755G0230. In the experimental validation, the PeMADS23-YFP fusion protein was observed to be localized in the nucleus of the *Arabidopsis* protoplast, while the PH01002755G0230-YFP fusion protein was observed in the cytoplasm (Fig. 2). Thus, the experimental results corroborated the predictions. Additionally, the K-domain appears to be essential for a MADS-box gene to gain entry into the nucleus. Compared with those of the BambooGDS, the sequences obtained using our protocols are more accurate, which will assist further MADS-box gene functional analyses.

**Table 1:** List of 42 MADS-box genes identified in *P. edulis* and their homologs in *Oryza* (aa, amino acids)

Name	Gene ID	Homologous	Length (aa)	Intron	Gene location	Subfamily	Predicted gene type
*PeMADS1*	PH01002127G0260	*OsMADS3*	266	7	PH01002127:173109–184,410(− stand)	Type II	AG-like
*PeMADS2*	PH01000306G0610	*OsMADS18*	257	7	PH01000306:378556–403,756(− stand)	Type II	FUL3 AP1 SQUA-like
*PeMADS3*	PH01000606G0250	*OsMADS14*	201	6	PH01000606:177440–184,284(+ stand)	Type II	FUL1 AP1 SQUA-like
*PeMADS4*	PH01000466G0340	*OsMADS68*	370	10	PH01000466:212382–216,357(− stand)	Type II	MIKC*
*PeMADS5*	PH01000437G0930	*OsMADS22*	228	7	PH01000437:618016–625,547(− stand)	Type II	SVP-like
*PeMADS6*	PH01000059G1270	*OsMADS56*	222	6	PH01000059:749594–811,731(+ stand)	Type II	TM3-like
*PeMADS7*	PH01000759G0450	*OsMADS56*	226	6	PH01000759:309532–372,975(+ stand)	Type II	TM3-like
*PeMADS8*	PH01000038G1550	*OsMADS47*	230	7	PH01000038:1113162–1,119,265(− stand)	Type II	SVP-like
*PeMADS9*	PH01000317G0080	*OsMADS2*	209	6	PH01000317:51918–54,493(+ stand)	Type II	PI GLO-like
*PeMADS10*	PH01001878G0200	*OsMADS4*	209	6	PH01001878:118831–120,592(+ stand)	Type II	PI GLO-like
*PeMADS11*	PH01000338G0120	*OsMADS31*	226	4	PH01000338:109773–112,174(+ stand)	Type II	GGM13-like
*PeMADS12*	PH01000616G0020	*OsMADS33*	190	5	PH01000616:15256–18,269(− stand)	Type II	AGL12-like
*PeMADS13*	PH01002743G0050	*OsMADS18*	257	7	PH01002743:23635–31,559(− stand)	Type II	FUL3 AP1 SQUA-like
*PeMADS14*	PH01000006G0210	*OsMADS20*	281	6	PH01000006:135925–141,334(− stand)	Type II	SQUA-like
*PeMADS15*	PH01005177G0100	*OsMADS26*	211	5	PH01005177:60648–64,034(+ stand)	Type II	AGL12-like
*PeMADS16*	PH01000794G0210	*OsMADS4*	273	5	PH01000794:136094–138,076(− stand)	Type II	PI GLO-like
*PeMADS17*	PH01001113G0580	*OsMADS32*	301	6	PH01001113:351365–358,010(− stand)	Type II	PI GLO-like
*PeMADS19*	PH01001303G0110	*OsMADS37*	183	5	PH01001303:60303–74,009(− stand)	Type II	OsMADS37-like
*PeMADS20*	PH01001952G0230	*OsMADS1*	244	7	PH01001952:162071–190,513(− stand)	Type II	AGL2-like SEP
*PeMADS21*	PH01000117G1210	*OsMADS37*	158	2	PH01000117:863423–865,146(− stand)	Type II	OsMADS37-like
*PeMADS23*	PH01002755G0230	*OsMADS50*	215	6	PH01002755:82368–142,165(− stand)	Type II	TM3-like
*PeMADS24*	PH01000080G0200	*OsMADS55*	259	7	PH01000080:117443–126,410(− stand)	Type II	SVP-like
*PeMADS25*	PH01003178G0150	*OsMADS65*	205	4	PH01003178:58826–64,955(− stand)	Type II	OsMADS37-like
*PeMADS26*	PH01000222G1060	*OsMADS34*	216	6	PH01000222:703088–717,231(+ stand)	Type II	AGL2-like SEP
*PeMADS28*	PH01000222G1190	*OsMADS15*	192	6	PH01000222:177607–183,539(+ stand)	Type II	FUL2 AP1 SQUA-like
*PeMADS29*	PH01001278G0330	*OsMADS3*	229	6	PH01001278:214488–227,311(+ stand)	Type II	AG-like
*PeMADS31*	PH01021006G0010	*OsMADS3*	229	5	PH01021006:308–2511(+ stand)	Type II	AG-like
*PeMADS32*	PH01003236G0170	*OsMADS29*	251	4	PH01003236:150540–151,005(− stand)	Type II	GGM13-like
*PeMADS33*	PH01001174G0480	*OsMADS5*	228	5	PH01001174:303844–317,831(+ stand)	Type II	AGL2-like SEP
*PeMADS34*	PH01002152G0120	*OsMADS56*	221	6	PH01002152:73202–96,884(+ stand)	Type II	TM3-like
*PeMADS35*	PH01000107G0570	*OsMADS56*	152	4	PH01000107:455631–474,775(− stand)	Type II	TM3-like
*PeMADS40*	PH01001750G0200	*OsMADS58*	230	5	PH01001750:217480–232,134(+ stand)	Type II	AG-like
*PeMADS41*	PH01001188G0490	*OsMADS15*	259	7	PH01001188:311503–334,928(+ stand)	Type II	FUL2 AP1 SQUA-like
*PeMADS43*	PH01000077G1380	*OsMADS55*	224	6	PH01000077:896999–903,795(+ stand)	Type II	SVP-like
*PeAP*_*3*_	N/A	*OsMADS16*	200	7	PH01001272:176122–180,192(+ stand)	Type II	AP_3_ GLO-like
*PeAGL6*	N/A	*OsMADS6*	240	4	PH01002217: 12110–41,791(+ stand)	Type II	AGL6-like
*PeMα1*	N/A	*OsMADS73*	97	0	PH01010177:5039–5332(+ stand)	Type I	Mα
*PeMα2*	N/A	*OsMADS73*	98	0	PH01000557:531750–532,199(− stand)	Type I	Mα
*PeMα3*	N/A	*OsMADS78*	212	0	PH01000604:455236–455,874(− stand)	Type I	Mα
*PeMα4*	PH01000954G0350	*OsMADS72*	377	1	PH01000954:196706–198,735(− stand)	Type I	Mα
*PeMα5*	N/A	*OsMADS75*	184	0	PH01002999: 94340–94,894(+ stand)	Type I	Mα
*PeMα6*	N/A	*OsMADS73*	87	0	PH01224566:45–309 (− stand)	Type I	Mα

**Table 2:** The detail information about these 17 genes before and after bioinformatics corrections

Gene ID	Imcomplete Reason	Original Length (aa)	Corrected Length (aa)
*PeMADS13*	Lack of K-domain	124	257
*PeMADS19*	Lack of K-domain	132	183
*PeMADS20*	Lack of K-domain	79	244
*PeMADS21*	Lack of K-domain	152	258
*PeMADS23*	Lack of K-domain	105	215
*PeMADS25*	Lack of K-domain	86	205
*PeMADS26*	Prior 319 bp is APO-domain	160	216
*PeMADS28*	Lack of M-domain	235	192
*PeMADS29*	Lack of M-domain	200	229
*PeMADS31*	Lack of M-domain	152	229
*PeMADS32*	M-domain is truncated	179	251
*PeMADS33*	Prior 184 bp is incorrect	181	228
*PeMADS34*	Prior 100 bp is incorrect	202	221
*PeMADS35*	Lack of M-domain	309	152
*PeMADS40*	Lack of M-domain	273	230
*PeMADS41*	Lack of M-domain	173	259
*PeMADS43*	Lack of M-domain	165	224

### Phylogenetic and conserved motif analyses of *PeMADS*s

**Fig. 3:**
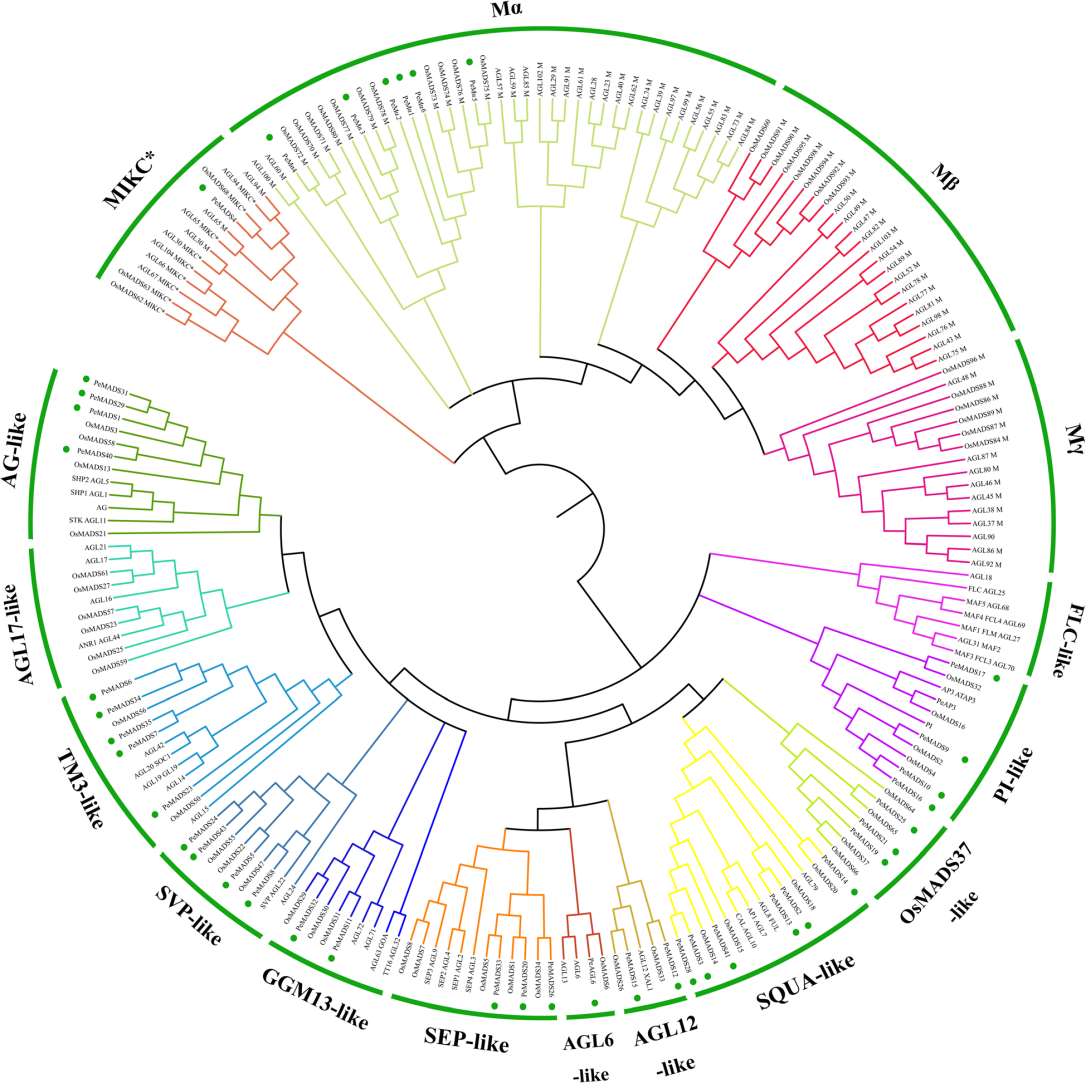
Phylogenetic analysis of MADS-box genes in *Arabidopsis*, *Oryza* and *P. edulis*. A total of 188 type II MADS-box amino acid sequences were used to construct the NJ tree. Bamboo MADS-box proteins are marked by green circles. The names of MADS-box genes from other species are based on previous studies: AGL: *Arabidopsis*; Os: *Oryza*; Pe: *P. edulis*

A phylogenetic tree of both type-I and type-II MADS-box genes was constructed to determine the evolutionary relationships between *P. edulis* MADS-box genes and the known MADS-box genes of *Arabidopsis* and *Oryza*. The exhibited groupings of MADS genes in moso bamboo were similar to those of the established model plants and can be further divided into 16 clades (Fig. 3). *PeMADS*s, except for the *AGL17*, *FLC*-like, and *Mβ-* and *Mγ* subgroups, formed 11 subgroups. Most *PeMADS*s belong to the MIKC^C^ type: two *P. edulis* orthologs each were present for the *Arabidopsis* GGM13and *AGL12* clades; three were present for the *AGL2*(*SEP*) and *OsMADS37* clades; four were present for the *AG* and *Solanum tuberosum* MADS11(*SVP*) clades; five were present for *GLO* (PI)-like, and six were present for *Mα*, *TM3*, and *SQUA*-like clades. Only one MIKC*-type gene, *PeMADS4*, has been identified in *P. edulis*. It has a longer I-domain, which appears to be the most prominent characteristic of the MIKC*-type proteins [49].

**Fig. 4:**
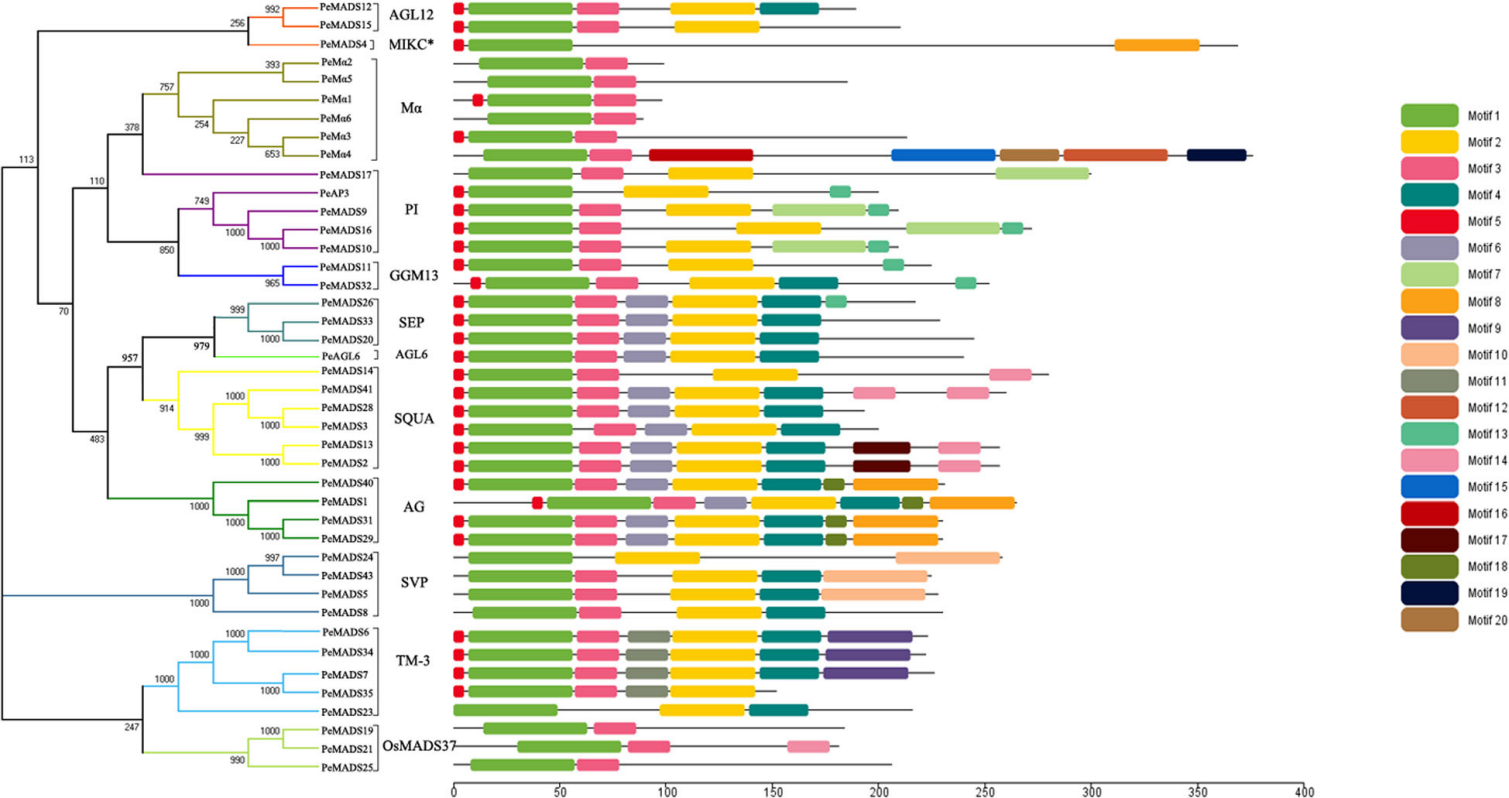
Distribution of conserved motifs in *P. edulis* MADS-box proteins identified by the MEME. Twenty putative conserved motifs were identified in bamboo MADS-box proteins by the MEME motif search tool. Different motifs are represented by different colors. The phylogenetic tree of all PeMADS members and the combined *p*-value from different groups are shown on the left side of the figure. Detailed information on each motif is presented in S4 Table

The motif distribution in PeMADS proteins was analyzed using the MEME program. The MEME software identified 20 conserved motifs in PeMADS, as well as their distribution (Fig. 4). The detected motifs were annotated using SMART protein analyzing software. Motifs 1 and 5 contain the typical MADS-box domain. Motifs 2 and 4 contain the second most conserved signature motif, the K-domain. Motifs 3, 6 and 18 contain the I-domain. C-terminals were the least conserved region of the MIKC proteins and had the most unknown motifs.

### Long-terminal repeat–retrotransposon (LTR–RT) analysis in long introns of *PeMADS*s

The CDSs and corresponding genomic DNA sequences of the *PeMADS*s were used to identify the gene structures of the *PeMADS* genes, with four (*PeMADS1*, − *2*, − *6*, and − *7*) having extraordinary long introns. In fact, those of *PeMADS*6 and *−* 7 are ~ 60 kb. We analyzed potential repeats in the *PeMADS* gene family using Repeat Masker online software [50] (Additional file 1: Table S3). The four above-mentioned MADS-box genes have a number of repeats in their intron areas. After analyzing these repetitive sequences, 140 transposable elements (TEs) were identified (Additional file 1: Table S4). LTR-RTs were the most abundant class, with 44 LTR/*Gypsy* and 26 LTR/*Copia* (26) superfamily members. In addition to LTR-RTs, DNA transposons, including DNA/*hAT-Tag1*, DNA/*Merlin*, and DNA/*Harbinger*, were found in these MADS-box genes.

### *cis*-element analysis of MADS-box family gene promoters and intron sequences

**Fig. 5:**
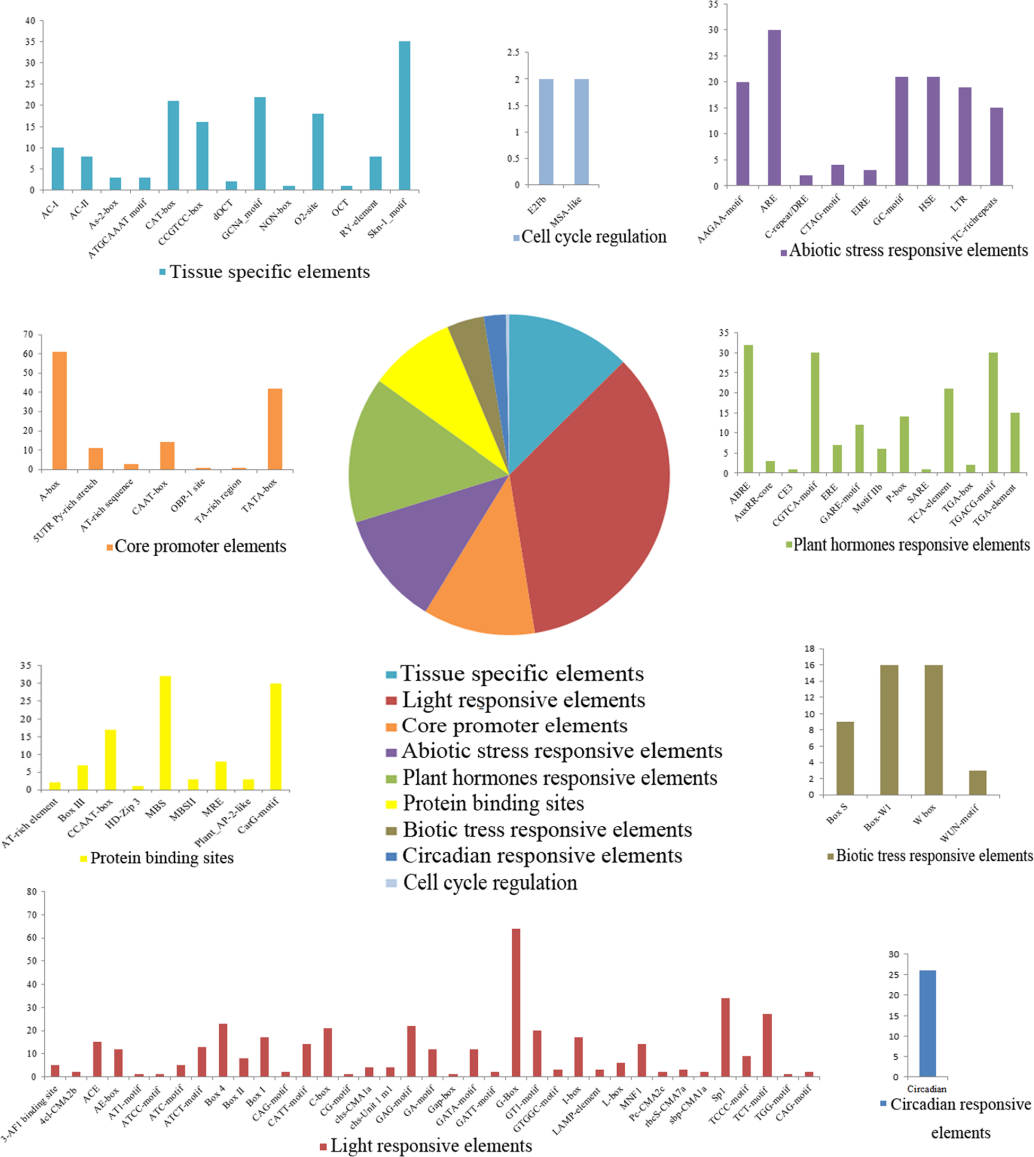
Frequency of cis-regulatory elements in the 1.5 kb upstream regions of *PeMADS*. The pie chart depicts the categorized nine types of cis-regulatory elements, and the corresponding colored bar chart represents the occurrence of different cis-elements

Most of the well-characterized MADS-box genes contribute to the processes of plant growth and responses to hormones or environmental stimuli, such as photoperiod and temperature. Determining the promoter region features of *PeMADS*s will help us to understand the expression patterns of bamboo MADS-box genes. To identify *cis*-regulatory elements in *PeMADS* genes, we extracted the promoter and intron regions for each *PeMADS* gene from the *P. edulis* genome and analyzed them using the PlantCare server. We categorized all of the *cis*-elements into nine broad categories based on their responsiveness to environmental stimuli. Details of the *cis*-elements are found in Additional file 1: Table S6. In the pie chart (Fig. 5), the proportion of light-responsive elements is the greatest, followed by tissue-specific, plant hormone-responsive, abiotic stress-responsive, and core promoter elements. G-box, Sp1, and TCT-motifs were the most frequently identified light-responsive elements. We identified several hormone-responsive *cis*-elements, such as ABRE, CGTCA-motif, TGACG, and TCA-elements, and abiotic stress-responsive elements, such as ARE, AAGAA-motif, LTR, HSE, GC-motif and TC-rich repeats. Most genes contain the endosperm expression elements Skn-1 and GCN4-motif. Some contain the meristem tissue-specific elements CAT and CCGTCC-box. Furthermore, we found some protein-binding sites in *PeMADS* promoter regions. The most numerous binding sites are MBS, CArG, and CCAAT-box. Previous studies showed that MADS-box proteins regulate the expression of target genes by binding to CArG motifs in their promoter regions [51]. CArG-motifs are not specific to promoter regions, but can also occur in the intron area. For instance, the *Arabidopsis AG* gene has a CArG-motif in its 1st intron [52, 53]. In our study, CArG motifs existed many times throughout *PeMADS* promoters and introns (Additional file 1: Table S7). The presence of these core binding sites indicated that PeMADS proteins form either homodimers or heterodimers to bind to the CArG-box sequence and affect the floral transition. The presence of the plant AP2-like-binding element implies that the three genes *PeMADS13*, *24*, and *43* can respond to endogenous floral developmental signals. This prediction is consistent with *PeMADS13*, *24*, and *43* showing relatively greater expression levels in floral tissues than in leaf tissues.

### Expression analysis of MADS-box genes during moso bamboo floral development

**Fig. 6:**
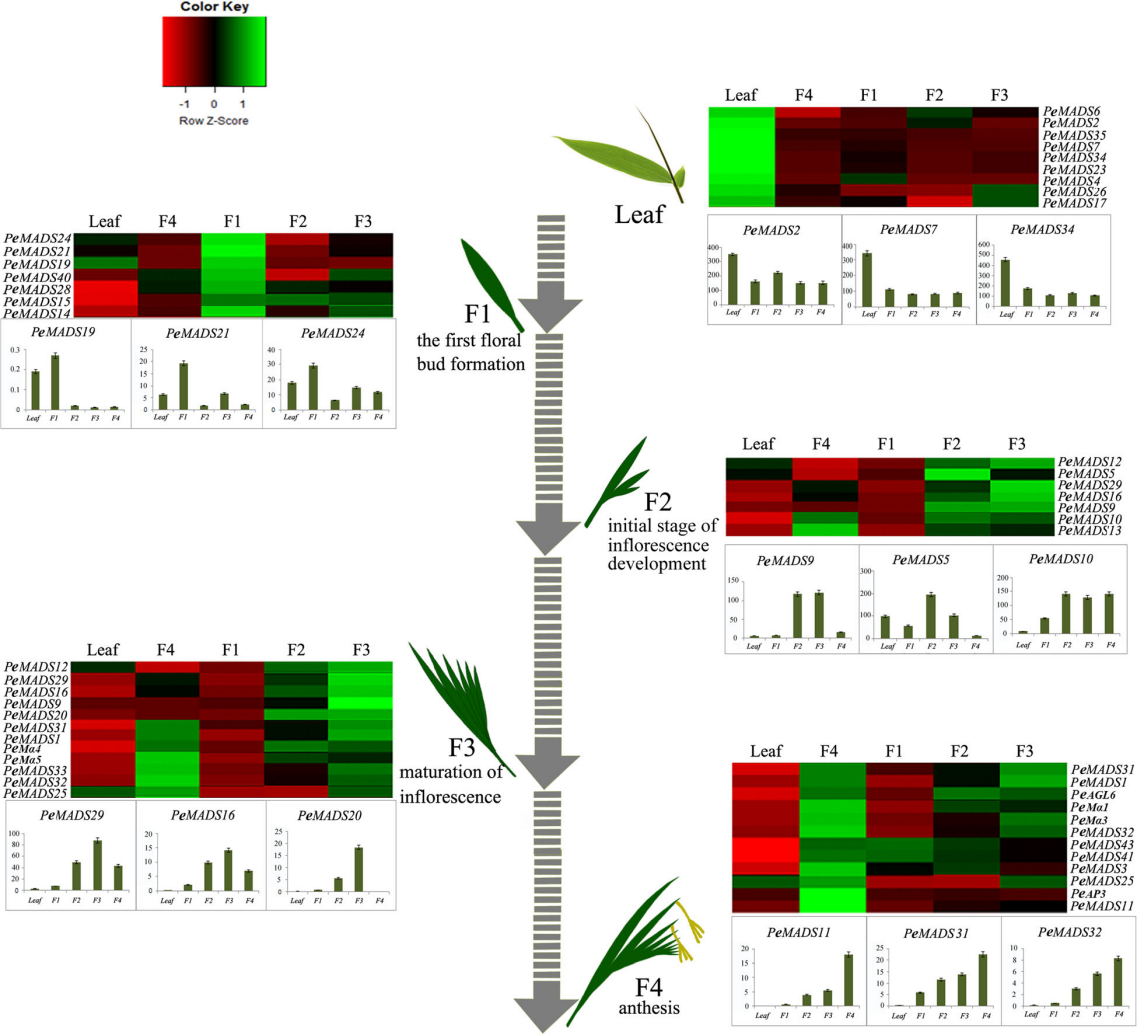
Hierarchical clustering of MADS-box gene expression in bamboo floral and leaf tissues. Results of real-time qPCR were calculated by the 2-ΔΔCt method using the Bio-Rad CFX Manager software (version 2.3). To normalize the variance among different floral samples, NTB was used as the housekeeping control. The expression levels were further normalized to those of the leaf sample. Mean values and SDs were obtained from three biological and three technical replicates. Red represents the low expression, black shows moderate expression, and green signifies high expression

We investigated the spatial expression patterns of the MADS-box genes in bamboo leaf tissues and in the different stages of floral development. *PeMADS*s were widely expressed in almost all of the tested tissues. For instance, *PeMADS2*, *− 3*, *− 6*, *− 13*, and − *17* were expressed in both vegetative and reproductive samples (Additional file 3: Figure S2; Additional file 1: Table S8). The relative expression levels of *PeMADS1*, *2*, *3*, *5*, *6*, *7*, *24*, *31*, *34*, *35*, and *41* were from 1.5- to 10-fold greater than that of the bamboo housekeeping gene *NTB* [44]. However, the transcripts of five genes, *PeMADS4*, *− 8*, *− 26*, *− 27*, *− 40*, and *PeMα2*, could not be detected in any sample. The expression patterns of the *PeMADS*s are shown as a schematic in Fig. 6. Most of the MADS-box genes were more highly expressed in the floret than in leaf. The transcript levels of *PeMADS3*, *5*, *16*, *19*, and *10* were from 2- to 5-fold greater in panicle than leaf, which indicated that these MADS-box genes play crucial roles both in floral transition and floral organ formation. The *PeMADS2*, *− 7*, *− 8*, *− 23*, *− 34*, and − *35* genes showed relatively lower expression levels in floral tissues than in leaf tissues, which suggested that these genes are involved in other processes of plant development.

Bamboo inflorescence development can be divided into four stages, F1–F4, from first floral bud to anthesis. *PeMADS14*, *− 15*, *− 19*, *− 21*, *− 24*, *− 28*, and *− 40* were mainly expressed at the F1 stage. *PeMADS2*, *− 5*, *− 9*, *− 10*, *− 16*, *− 20*, and *− 29* were prominently expressed at the F2 and F3 stages. Then, the expression levels of *PeMADS1*, *− 3*, *− 9*, *− 25*, *− 32*, and *− 33* were greater higher in the F4 stage. In general, bamboo MADS-box genes within subfamilies share relatively similar expression patterns. Based on the expression patterns (Fig. 6), MADS-box genes, such as *SQUA*-*like* (*PeMADS13*, *− 14*, and *− 28*) and *OsMADS37*-*like* genes (*PeMADS19* and *− 21*) were found to be involved in floral meristem activity and were highly expressed at the F1 stage. The “ABCDE” model genes *AGL2*-like (*PeMADS20* and *− 33*), *GLO*-like (*PeMADS9*, *− 10*, and *− 16*), and *AG*-like (*PeMADS1*, *− 29*, and *− 31*) were up-regulated during floral-organ development. Homologs of the *SVP/StMADS11*-like subfamily are hallmark meristematic genes, and *PeMADS5*, *− 8*, *− 24*, and − *43* displayed their greatest expression levels in F2, leaf, F1, and F4, respectively. The type-I genes *PeMα1*, *− 3*, *− 4*, and *− 5* were highly expressed during the late developmental stages of the bamboo inflorescence. *PeMADS5* was greatly expressed in the F2 stage, and its expression decreased with the development of the bamboo inflorescence, suggesting a role for this gene in spikelet initiation.

### Overexpression of *PeMADS5* in wild-type *Arabidopsis* plants causes an early flowering time and abnormal floral morphology

**Fig. 7:**
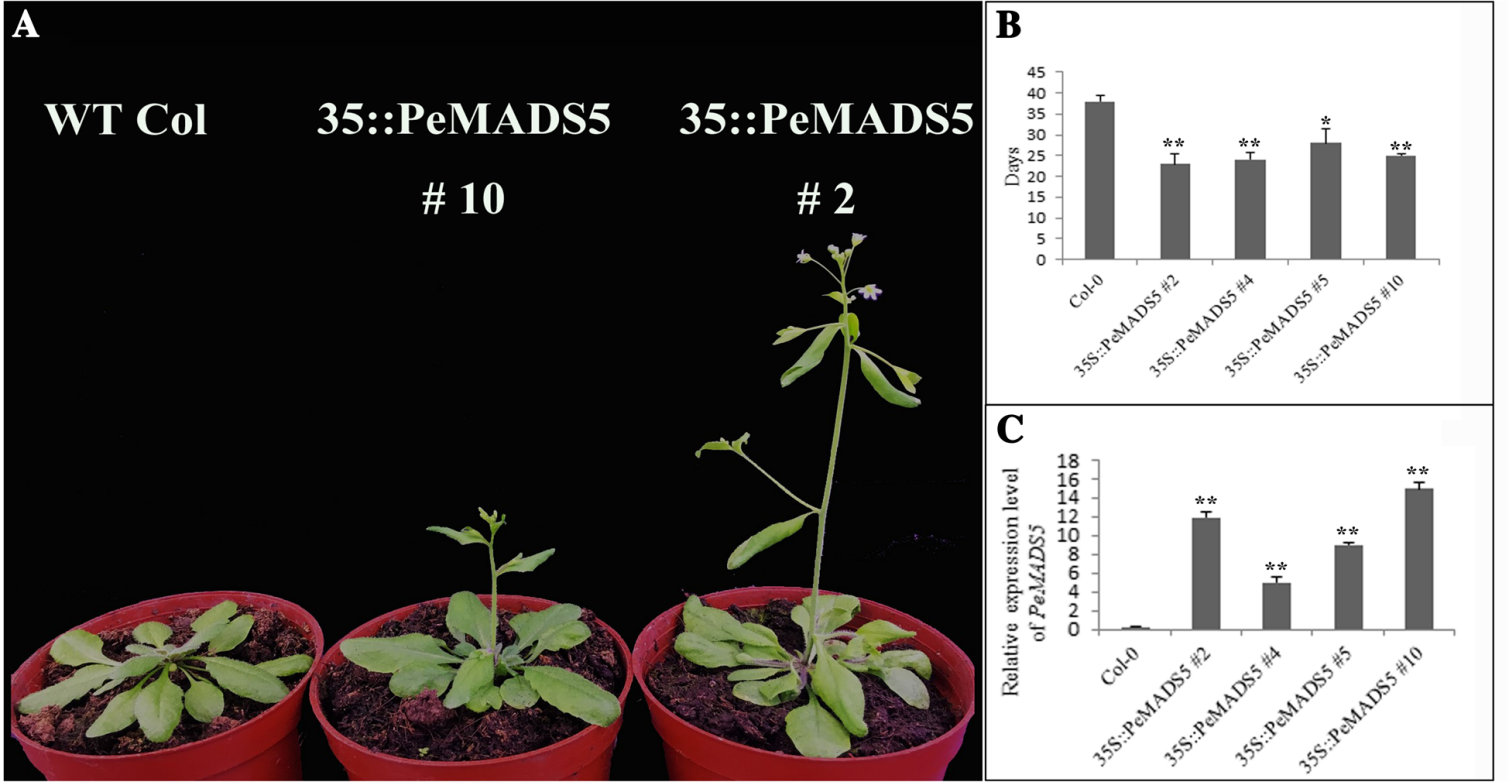
Ectopic expression of *PeMADS5* causes early flowering in *Arabidopsis*. **a** The flowering phenotype of *PeMADS5* transgenic *Arabidopsis* plants under LD conditions. **b** Flowering time was measured under LD conditions. Error bars on each column indicate SD from three replicates. **c** *PeMADS5* expression levels in col-0 and four 35S:: *PeMADS5* transgenic lines by qRT-PCR, with the Actin2 gene as an internal control. The *means Significant difference at *P* ≤ 0.05 compared with the wild-type by Student’s test, and ** means the difference at *P* ≤ 0.01 with wild-type

**Fig. 8:**
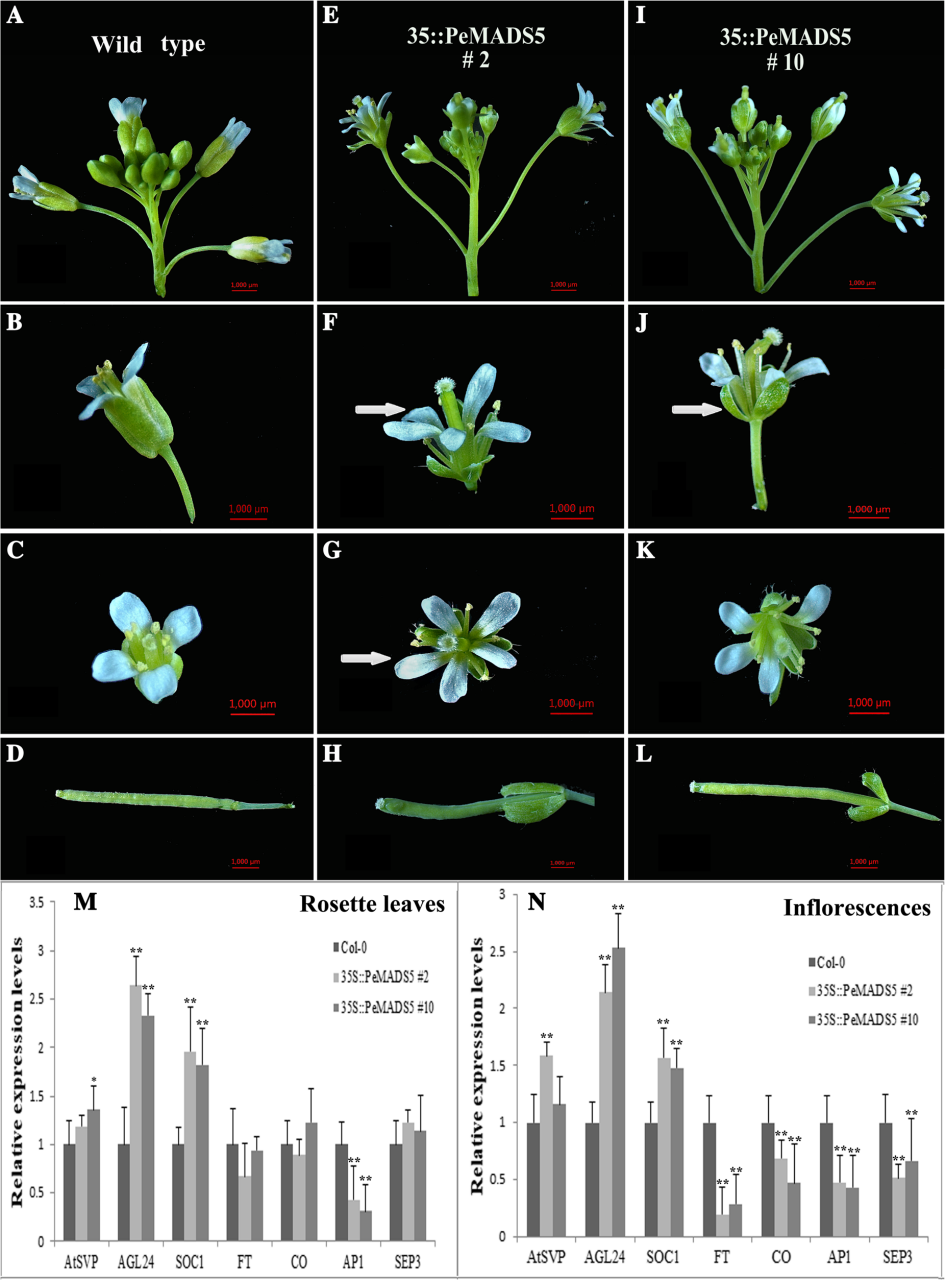
Morphological analysis of 35S::*PeMADS5* transgenic *Arabidopsis*. Light micrographs of Wild-type *Arabidopsis* Col-0 (**a**–**d**) and 35S::*PeMADS5* transgenic plants (#2: E to H; #10: I to L). **a** Wild-type *Arabidopsis* Col-0 inflorescence. **b** Side view and (**c**) top view of a wild-type *Arabidopsis* Col-0 flower at anthesis. **d** A wild-type *Arabidopsis* matured fruit. **e** 35S::*PeMADS5* #2 (**i**) 35S::*PeMADS5* #10 inflorescences. **f** Side view and (**g**) top view of a 35S::*PeMADS5* #2 flower at anthesis. Note the extra petal indicated with an arrow. **j** Side view and (**k**) top view of a 35S::*PeMADS5* #10 flower at anthesis. Note the sepals with leaf-like characteristics and did not completely enclose the inner developing organs. Both 35S::*PeMADS5* #2 (**h**) and #10 (**l**) matured fruits have attached petals denoted with arrows. Relative expression levels of different flowering time genes (*SVP*, *AGL24*, *SOC1*, *FT*, *CO, AP1* and *SEP3*) in both leaves (**m**) and flowers (**n**) as determined by qRT-PCR, with the Actin 2 gene as an internal control. Error bars on each column indicated from three replicates. The * indicates Significant difference at P ≤ 0.05 compared with the wild-type by Student’s test, and ** means the difference at P ≤ 0.01 with wild-type

Genes from *SVP/StMADS11*-like subgroup are involved in panicle branching, flowering time determination, and floral meristem specification in *Arabidopsis* and *Oryza*. In this study, we found four *SVP/StMADS11*-like genes, *PeMADS5*, − *8*, − *24*, and − *43*, in bamboo. Among them, *PeMADS5* was expressed highly during inflorescence initiation. We selected this gene for the functional studies. We obtained 12 single-copy independent transgenic lines for *PeMADS5*. Flowering time was determined in the T3 generation under the long-day conditions, and *35S::PeMADS5* plants flowered significantly earlier than wild-type plants (Fig. 7a and b). Four of the transformed *Arabidopsis* T3 lines (*35S::PeMADS5* #2, #4, #5, and #10) showed various degrees of phenotypic alterations in their reproductive organs compared with wild-type plants (Fig. 8 and Additional file 4: Figure S3). To confirm the overexpression of transgenes, *PeMADS5* transcript levels were analyzed by RT-PCR in these four transgenic lines (Fig. 7c). Relative *PeMADS5* expression levels in 35S::*PeMADS5* #2 and #10 were greater than in other lines, and they also had ‘strong’ phenotypes as shown in Fig. 8e, f. Flowers of 35S::*PeMADS5* #2 and #10 exhibited sepals that formed leaf-like structures and did not completely enclose the inner developing organs (Fig. 8f and j). Additionally, 35S*: PeMADS5* #2 had five petals instead of four (Fig. 8g). Furthermore, sepals remained attached in fruit of both transgenic lines #2 and #10 (Fig. 8h and l), which was in contrast to wild-type *Arabidopsis* plants (Fig. 8d). In addition to the phenotypic analysis, the expression levels of some flowering-related genes, including those involved in flowering time and flower organ development, were also analyzed (Fig. 8m and n). In both rosette leaves and inflorescences, *AGL24* and *SOC1* transcripts were significantly upregulated in 35S::*PeMADS5* #2 and #10 compared with wild-type, while *AP1* was downregulated. The expression levels of *FT* and *SEP3* in 35S::*PeMADS5* were nearly 0.3- and 0.5-fold, respectively, those of the wild-type inflorescences, while they were expressed at slightly different levels in leaves. Thus, *PeMADS5* may act as a floral activator in bamboo flowering.

### Interaction of PeMADS5 with other floral-related MADS-box proteins

**Fig. 9:**
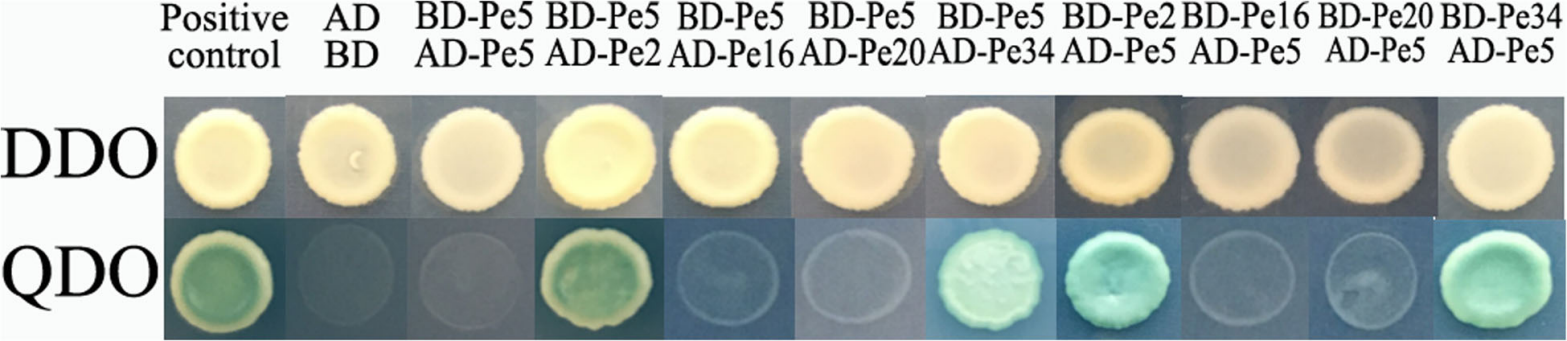
Yeast two-hybrid assays of PeMADS5 and its interacting proteins. The *PeMADS5*, *PeMADS2*, *PeMADS16*, *PeMADS20* and *PeMADS34* genes were fused both in-frame to the GAL4 DNA-binding domain (BD)-coding sequences and activation domain (AD)-coding sequences. Cell growth on -Leu-Trp dropout selective medium (-DDO) represents normal cells (upper panel) and -Leu-Trp-His-Ade dropout selective medium (-QDO) represents positive interactions (bottom panel). Pe refers to PeMADS

In *Arabidopsis*, AGL24 interacts with SOC1 and FUL in the shoot apical meristem to promote flowering [54]. During floral organ formation, AGL24–AP1 dimers can also interact with the class B gene *PISTILLATA* (*PI*) and the class E gene *SEPALLATA* (*SEP*) [55, 56]. Therefore, we performed a yeast two-hybrid assay using the protein-coding regions of *PeSOC1* (*PeMADS34*), *PeAP1* (*PeMADS2*), *PeSEP3* (*PeMADS20*), and *PePI* (*PeMADS16*) to obtain insights into the interaction patterns of the PeAGL24 (*PeMADS5*) protein in bamboo. The coding sequences of *PeMADS2*, *− 5*, *− 16*, *− 20*, and − *34* were fused to the binding and activating domains and their abilities to interact were determined (Fig. 9). PeMADS5 (AGL24) could interact independently with PeMADS34 (SOC1) and PeMADS2 (AP1), but not with PeMADS16 (PI) or PeMADS20 (SEP3).

## Discussion

### A practical alternative way to obtain a complete gene sequence

Owing to the current assembly of the bamboo genome, some *PeMADS* sequences were incorrectly annotated and lack full-length CDSs. For instance, some MADS-box genes identified in the BambooGDS lack the characteristic M- or K-domain. In this study, we employed a transcriptome assembling and polishing method to achieve more accurate MADS-box sequences in bamboo. Briefly, we first used the truncated *PeMADS* sequences as query seeds to seek optimally aligned short reads. These reads extended both the 5′- and 3′-end regions until full-length CDSs with 5′ UTRs and 3′ UTRs were achieved. By cloning full-length genes and validating protein subcellular localizations, we demonstrated that this bioinformatics procedure reliably produced full-length MADS-box gene sequences.

### MADS-box type-I subfamily members may be lost during *Phyllostachys*’ evolution

A total of 36 MIKC-type MADS-box genes in moso bamboo were identified in this study. This number is similar to that in *Arabidopsis* (39), *Oryza* (38), and other well-studied species (Table 3). Non-candidate genes belonging to the type-I MADS-box were found. Using *Oryza* and *Brachypodium* type-I genes sequences as query, only six Mα genes have been found. In gymnosperms, type-I MADS-box genes are underrepresented [57]. Only two type-I MADS-box genes are present in the most recent common ancestor of seed plants [58]. Although the number of type-I MADS-box genes appeared to have dwindled in some angiosperms, including wheat, poplar, and barley, we observed an extreme decrease in bamboo [46, 59, 60]. *Brachypodium* has the same number of type-II genes as bamboo but also has fewer members of the type-I subfamily (Table 3). Therefore, the common ancestor of bamboo and *Brachypodium* may have undergone a gene loss event after it initially diverged from *Oryza* 45–50 million years ago. Subsequently, we hypothesize that bamboo experienced a second gene loss event after speciation. The expansion and construction of orthologous clusters showed the greatest number, among the grass family members, in bamboo after species divergence [61]. Thus, gene families in bamboo are more divergent and polarized than in other grass species. Another hypothesis for the lack of type-I genes is based on numerous scaffolds not being obtained by the *Phyllostachys* whole-genome shotgun sequencing, resulting in the lack of assembled pseudochromosomes. Given the presence of small fragments in the assembly, the lack of type-I subfamily members may be a statistical riddle because of the imperfect genome sequence [61]. Conversely, orthologous type-I genes may exist in bamboo but were not identified because of the limited number of phylogenetically informative sites. In summary, bamboo may have a unique type-I subfamily evolutionary pattern but share a common type-II subfamily evolutionary pathway with other angiosperms.

**Table 3:** Total number of MADS-box genes within each group among nine species

	Mα	Mβ	Mγ	I type	MIKC^C^	MIKC^*^	II type	Pseudogenes	Total
*P. edulis*	6			6	36	1	36	8	42
*Arabidopsis*	25	20	16	61	39	8	45		107
*Oryza*	13	9	10	32	40	3	43		75
Maize	27	3	2	32	39	4	43		75
*Sorghum*	26	2	2	30	33	2	35		65
*Physcomitrella patens*	2	2	3 (unclassified)	7	6	11	17	2 (1 Mβ; 1 MIKC^*^)	26
*Brachypodium*	9	7	2	18	32	7	39		57
Poplar	23	12	6	41	55	2		7 (Mδ)	105
Soybean	37	14	24	75	81	7	88		163

### Presence of long introns and the insertion of LTR-RT elements in the *Phyllostachys* MADS-box family

A notable characteristic of the structure of bamboo MADS-box genes is the presence of long introns. This is similar to findings in the repeat-rich genomes of *Vitis vinifera*, *Zea mays*, and Norway spruce [62–64]. An analysis of sequences containing long introns revealed that they all contain numerous repeats, suggesting that intron expansion in bamboo was promoted by repeat insertion. By contrast, exon size and the number of MADS-box genes were consistent with those of other well-studied species. Long introns did not influence expression levels (Fig. 6). TEs are the most abundant DNA components in higher eukaryotes [65], and is approximately 59% of the bamboo genome consists of TEs [61]. In the full-length genome sequences of *PeMADS1*, *− 2*, *− 6*, and *− 7,* 140 TEs were found. Most of the repeats could be assigned to known TE repeat families (Additional file 1: Table S5). LTR-RTs were the most abundant fraction of TEs, with the *Gypsy*-type LTR superfamily members being more abundant than *Copia*-type LTR superfamily members. This was consistent with the genome-wide analysis of LTR-retroelements in *P. edulis* [66]. These TEs are prone to insertion and/or maintenance in the intronic regions of MADS-box genes. Thus, *PeMADS*s, with their abundant repetitive sequences, may be hotspots for TE insertion. Large-genome species, such as maize, barley, and wheat, contain a large number of LTR-RT element that, which have been amplified within the last few million years [67, 68]. Therefore, we hypothesized that the accumulation of LTR-RTs in bamboo gene families may be a main factor that led to the increased genome size without polyploidization, like that identified in *Oryza australiensis*.

### Expression analysis of *Phyllostachys* MADS-box genes and their orthologs during inflorescence development

To gain insight into bamboo MADS-box gene expression patterns, we carried out a qPCR expression study using different floral tissues. The study revealed that critical regulatory genes for spikelet meristem development might be conserved between *Phyllostachys* and *Oryza*. For example, genes from the *AG*-like subfamily are essential for reproductive structure morphogenesis [69–71]. As shown in Fig. 6, bamboo *AG*-like genes *PeMADS1*, *− 29*, and *− 31* are primarily expressed in the later stages of floral development, whereas their *Oryza* orthologs *OsMADS3* and − *58* have relatively greater expression levels in reproductive tissues [18]. Several *SQUA*-like genes are proposed to be vital regulators in inflorescences or floral meristems and are involved in organ identity [72]. In the present study, *Phyllostachys SQUA*-like genes *PeMADS2*, *− 3*, *− 13*, and − *41* were significantly expressed at all stages of inflorescence development, with expression patterns parallel to those of their putative *Oryza* orthologs *OsMADS14*, − *15*, and − *18*, respectively [18]. Nevertheless, modifications in orthologous gene expression patterns may be necessary owing to neofunctionalization and subfunctionalization. *OsMADS34*, a member of the *SEP* subgroup, was a key regulator of rice inflorescence and spikelet architecture. In rice, *OsMADS34* expression was detected in the floral meristem, and the *osmads34* mutants develop altered inflorescence morphology [73]. Additionally, *PeMADS26*, the *OsMADS34* homolog in bamboo, was highly expressed in leaves but not florets. Similarly, *PeMADS6* and − *34* from the TM3-like subgroup were predominantly expressed in the bamboo leaf but not in the spikelet meristems. In *Oryza*, *MADS56* showed a high transcript accumulation level at all stages, especially in panicle materials [18]. We surmised that the differences in inflorescence structures might be the result of expression pattern changes in the conserved genes of the grass family.

### *PeMADS5* may interact with *PeMADS34* and *PeMADS2* to control flowering time and floral organ development

*PeMADS5* belongs to the *StMADS11/SVP* clade of the MADS-box gene family, which has two diversified functional members in *Arabidopsis*: *SVP* and *AGL24* [74]. In *Arabidopsis*, *AGL24* acts as an integrator of multiple flowering signals and function together with *SOC1* to regulate the floral transition and inflorescence meristem identity [75, 76] *SVP* controls flowering time by negatively regulating the expression of *FLOWERING LOCUS T* (*FT*) by directly binding to the CArG motifs in the *FT* sequence [77]. The overexpression of *AGL24* in *Arabidopsis* results in early flowering and floral abnormalities, such as leaf-like sepals, or the transformation of floral meristems into inflorescence meristems [75, 76, 78]. In contrast, the overexpression of *SVP* results in late flowering and the loss of carpels, as well as the conversion of flowers into shoot-like structures [78]. The ectopic overexpression of *PeMADS5* in *Arabidopsis* triggered an earlier flowering time in all of the transgenic lines, and 4 out of the 15 lines developed aberrant flower phenotypes (Fig. 8). The expression level of *PeMADS5* was greater in transgenic lines with an obvious mutant phenotype, and proportionately lower in lines with less obvious mutant phenotypes (Fig. 7c). The phylogenetic analysis of *PeMADS5* revealed that it had a closer relationship to *OsMADS22* and *OsMADS55* (Fig. 3). The constitutive expression of *OsMADS22* and *OsMADS55* leads to floral reversion phenotypes, including leaf-like sepals, which is similar to the *PeMADS5*-overexpression phenotype, but *OsMADS55* acts as a floral repressor by suppressing the expression levels of *Hd3a* and *SOC1* [79]. In Arabidopsis, the identity of each floral organ has been determined by a specific combination of floral homeotic genes constituting the ABCDE model, in which the class E *SEP3* and class A *AP1* genes have critical roles [80–82]. Thus, the relatively low expression levels of *SEP3* and *AP1* (Fig. 8m and n) may affect normal floral development, leading to the abnormal floral phenotypes of the *35S::PeMADS5* plants. The ectopic expression of class C (*AG* subfamily) in *Arabidopsis* results in the conversion of petals to stamens and of sepals to carpels, as observed in *35S:AG* lines, and this is similar to the *35S:PeMADS5* phenotypes [83]. AP1–SVP and AP1–AGL24 dimers can bind CArG boxes in the second *AG* intron and affect the formation of floral organs [53]. It is likely that multiple mechanisms act on *PeMADS5* through class C, D and E genes. Furthermore, the yeast two-hybrid assay showed that the PeMADS5 protein interacts with PeMADS2 (PeAP1) and PeMADS34 (PeSOC1) proteins. This suggests that the interaction domains of the AGL24*,* SOC1, and AP1 proteins are conserved between *Arabidopsis* and bamboo. In conclusion, we found that *PeMADS5* is functionally similar to *AGL24*, that it may interact with PeSOC1 as an integrator of flowering inducers, and that it associates with PeAP1 to regulate flower development.

## Conclusion

The recent release of the *P. edulis* genome sequence enabled us to identify and comprehensively analyze its MADS-box family. We identified 42 full-length bamboo MADS-box genes, 6 that were type I and 36 that were type II. Consequently, we hypothesized a low formation rate and high destruction rate for type-I genes in *Phyllostachys*. The MADS-box genes and proteins of moso bamboo were classified and their evolutionary relationships with those of other eudicots were evaluated using phylogenetic and structural analyses. A survey of the expression levels of bamboo MADS-box genes in floral and leaf tissues revealed that some MADS-box genes are involved in inflorescence development. Most *Phyllostachys* MADS-box genes appeared to have similar expression patterns to those of their orthologous genes. MADS-box genes with specific expression patterns may play particular functions in bamboo floral development and can be considered candidate genes for cloning and further functional analyses. In addition, the overexpression of *PeMADS5*, a candidate gene from the *StMADS11* clade, in *Arabidopsis* caused early flowering and abnormal floral organ development. Therefore, further studies are required to understand how *PeMADS5* is regulated and whether and how it regulates other genes to control flower development in *Phyllostachys*.

## Additional files

Additional file 1: **Table S1.** List of primers used for *PeMADS* genes cloning. **Table S2.** List of primers used for qPCR in *P. edulis*. **Table S3.** List of repeat numbers in the *PeMADS* gene family identified by the Repeat Masker online software. **Table S4.** List of transposable elements in *PeMADS1, − 2, − 6*, and − *7*. **Table S5.** The primers for Real-time quantitatively RT-PCR in *Arabidopsis*. **Table S6.** List of cis-elements in 1.5 kb upstream promoter regions of *PeMADS*s. **Table S7.** List of CArG box in *PeMADS*s promoter and intron region. (XLSX 136 kb)

Additional file 2: **Figure S1.** The alignment of the cloned sequence, *PeMADS23*, and PH01002755G0230. (TIF 1624 kb)

Additional file 3: **Figure S2.** qPCR expression analysis of bamboo MADS-box genes in floral and leaf tissue (TIF 5256 kb)

Additional file 4: **Figure S3.** The phenotypes of *PeMADS5* transgenic *Arabidopsis* plants under LD conditions (A and B) and SD conditions (C). (TIF 2203 kb)

## Abbreviations

AG: AGAMOUS
AGL: AGAMOUS LIKE
AP: APETALA
cDNA: Complementary DNA
CFO1: Chimeric Floral Organs
DEF: DEFICIENS
FLC: Flowering Locus C
FUL: FRUITFULL
GLO: GLOBOSA
HMM: Hidden Markov Model
LTR-RTs: Long Terminal Repeat-Retrotransposons
MEF2: Myocyte Enhancer Factor 2
NCBI: National Center for Biotechnology Information
NJ: Neighbor-joining
NTB: Nucleotide Tract-Binding Protein
PCR: Polymerase chain reaction
qPCR: Quantitative Real-time Polymerase Chain Reaction
SEP: SEPALLATA
SHP: SHATTERPROOF
SOC: Suppressor of Overexpression of CONSTANS
SQUA: SQUAMOSA
SRF: Serum Response Factor
SVP: Short Vegetative Phase
TEs: Transposable Elements
TF: Transcription factor
