# Supplementary figures and images for: Genome-wide identification of MADS-box family genes in moso bamboo (Phyllostachys edulis) and a functional analysis of PeMADS5 in flowering
Source: BMC Plant Biol. 2018 Sep 3;18:176. doi: 10.1186/s12870-018-1394-2 (PMC6122543; doi:10.1186/s12870-018-1394-2)

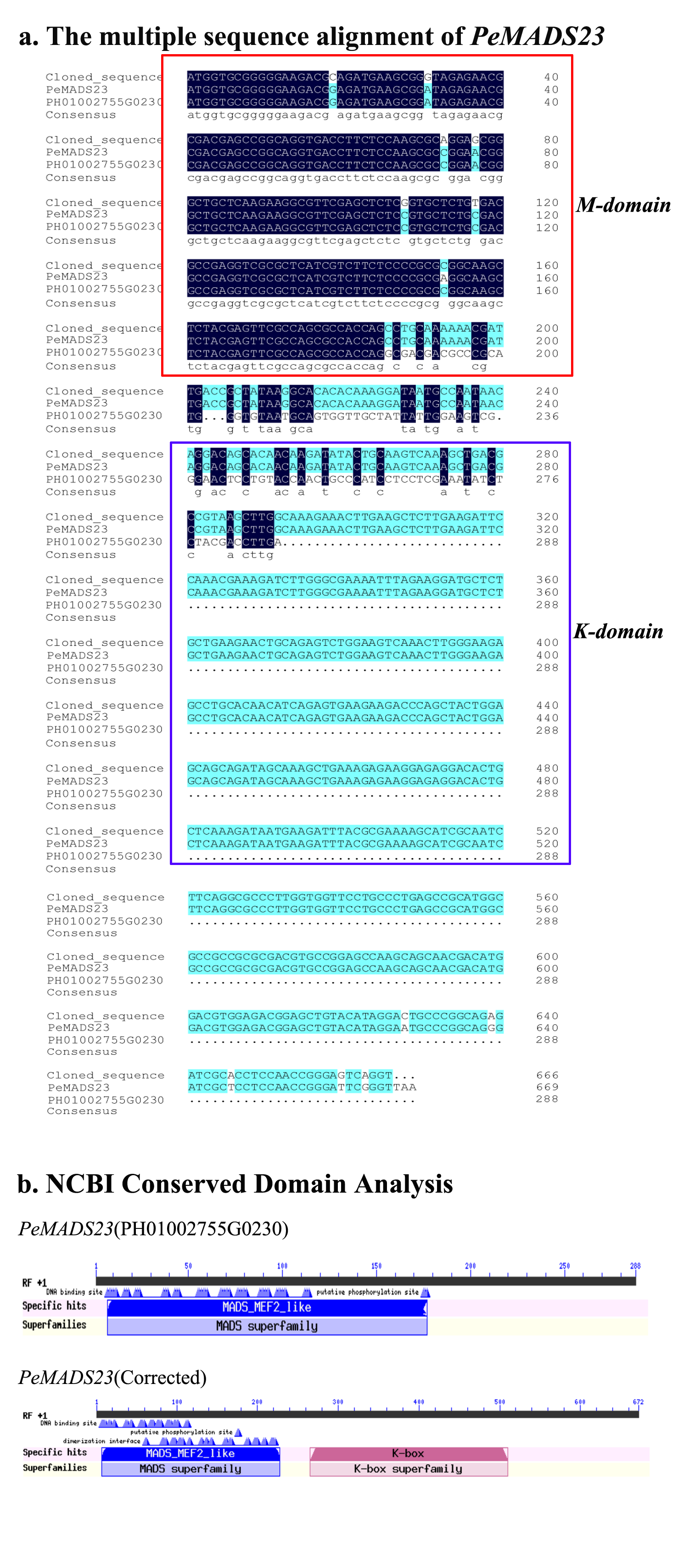

Supplement: Supplementary file 2 — Figure S1. The alignment of the cloned sequence, PeMADS23, and PH01002755G0230. (TIF 1624 kb) [file 12870_2018_1394_MOESM2_ESM.tif]

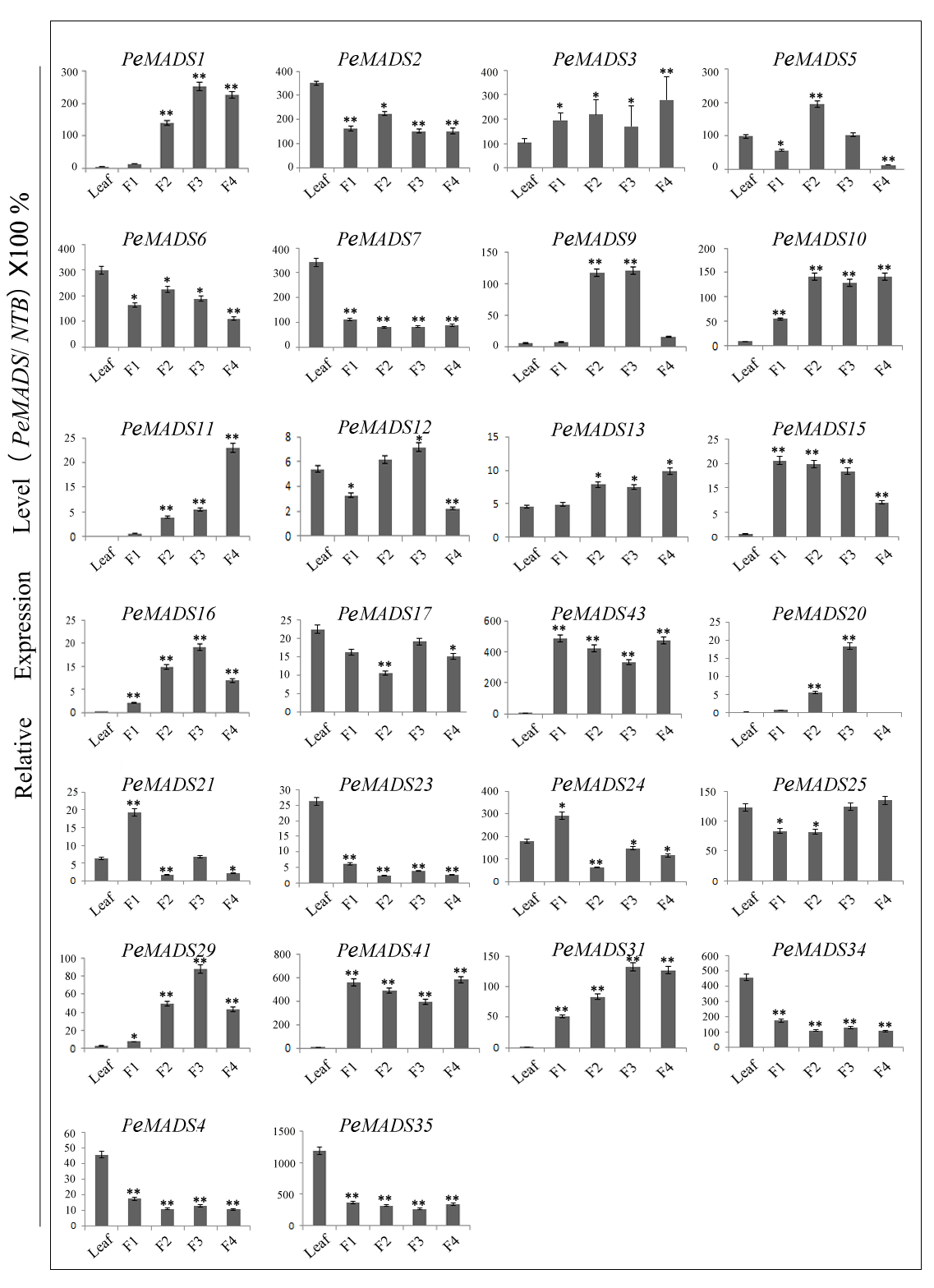

Supplement: Supplementary file 3 — Figure S2. qPCR expression analysis of bamboo MADS-box genes in floral and leaf tissue (TIF 5256 kb) [file 12870_2018_1394_MOESM3_ESM.tif]

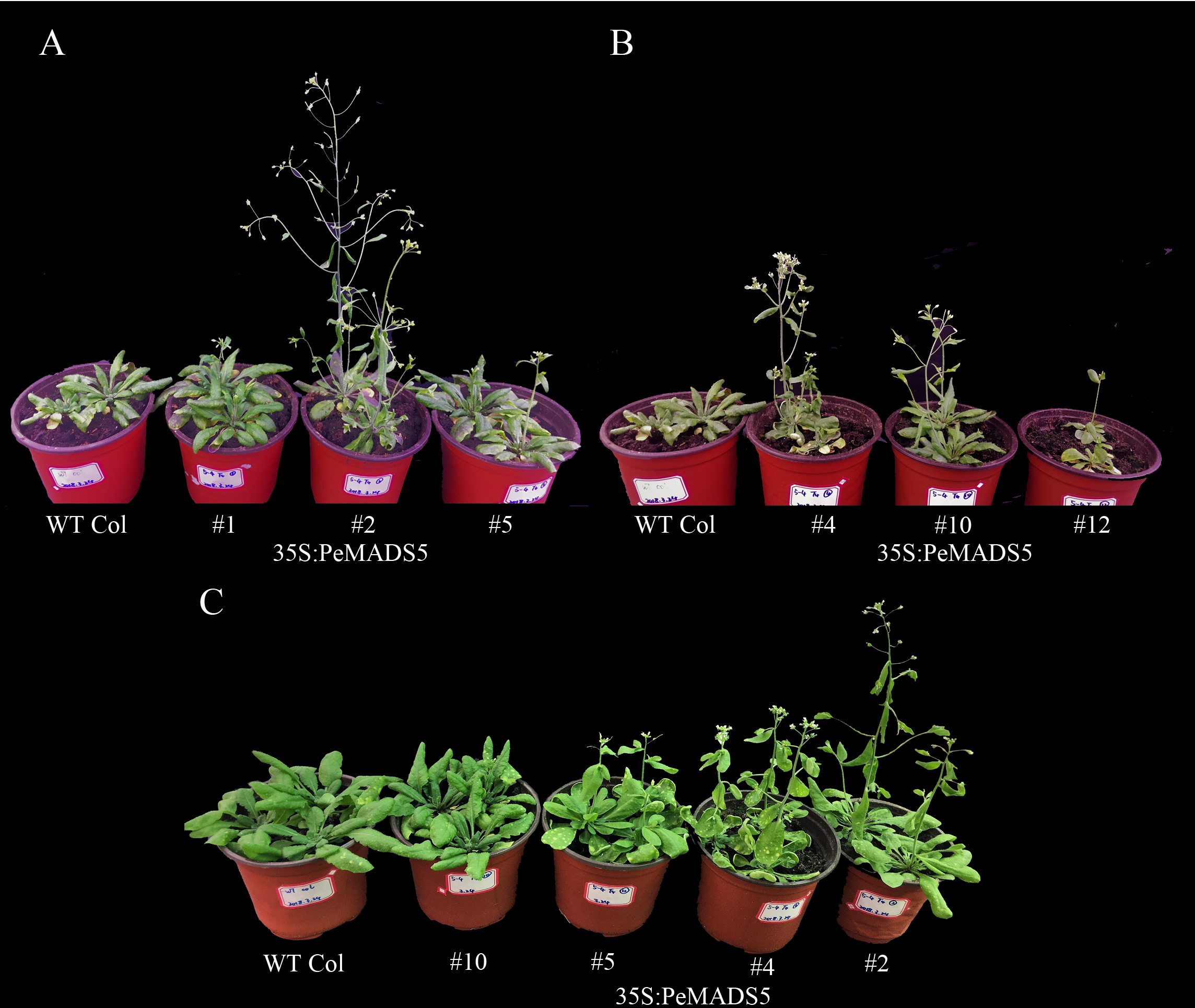

Supplement: Supplementary file 4 — Figure S3. The phenotypes of PeMADS5 transgenic Arabidopsis plants under LD conditions (A and B) and SD conditions (C). (TIF 2203 kb) [file 12870_2018_1394_MOESM4_ESM.tif]
